# The Lack of ADAM17 Activity during Embryonic Development Causes Hemorrhage and Impairs Vessel Formation

**DOI:** 10.1371/journal.pone.0013433

**Published:** 2010-10-15

**Authors:** Matthias Canault, Kaan Certel, Daphne Schatzberg, Denisa D. Wagner, Richard O. Hynes

**Affiliations:** 1 Immune Disease Institute, Boston, Massachusetts, United States of America; 2 Program in Cellular and Molecular Medicine, Children's Hospital Boston, Boston, Massachusetts, United States of America; 3 Department of Pathology, Harvard Medical School, Boston, Massachusetts, United States of America; 4 Howard Hughes Medical Institute, Chevy Chase, Massachusetts, United States of America; 5 David H. Koch Institute for Integrative Cancer Research, Massachusetts Institute of Technology, Cambridge, Massachusetts, United States of America; Leiden University Medical Center, Netherlands

## Abstract

**Background:**

ADAM17/TACE activity is important during embryonic development. We wished to investigate possible roles of this metalloprotease, focusing on vascular development.

**Methodology/Principal Findings:**

Mice mutant in the enzymatic activity of ADAM17 were examined at various stages of embryonic development for vascular pattern and integrity using markers for vessel wall cells. We observed hemorrhage and edema starting at embryonic day E14.5 and becoming more severe as development proceeded; prior to embryonic day E14.5, embryos appeared normal. Staining for PECAM-1/CD31 revealed abnormalities in the patterns of branching of the embryonic vasculature at E14.5.

**Conclusions/Significance:**

These abnormalities preceded association of pericytes or monocyte/macrophage cells with the affected vessels and, therefore, presumably arise from defects in endothelial function consequent upon failure of ADAM17 to cleave one or more substrates involved in vascular development, such as Notch, Delta, VEGFR2 or JAM-A. Our study demonstrates a role for ADAM17 in modulating embryonic vessel development and function.

## Introduction

The members of the ADAM (A Disintegrin And Metalloprotease) family of proteases are metzincin-enzymes that cleave a variety of substrates including cytokines, proteins of the extracellular matrix, cell adhesion molecules and growth factors. ADAM17, also referred to as Tumor necrosis factor Alpha Converting Enzyme (TACE, CD156b) [1], is the most studied member of this family and participates in the shedding of more than 40 different substrates [2]–[4]. ADAM17 is expressed in most tissues, where it plays important physiological and pathophysiological roles since it has been implicated in inflammatory disorders, brain pathology and cancer. In addition, ADAM17 activity appears to be important during embryonic development since mice lacking ADAM17 activity die during the late gestational stages or soon after birth [5]. These mice not only present defects in skin, muscle and neuronal tissues [5] but also exhibit impaired heart and lung morphogenesis [6], [7] that were postulated to be responsible for the premature mortality. Interestingly, an underdeveloped pulmonary vascular network was also observed in mice deficient in ADAM17 activity [7] suggesting a possible requirement for a fully functional ADAM17 for normal angiogenesis in embryos. In vitro, potential involvement of ADAM17 in angiogenesis was recently illustrated in two studies where knocking down [8] or silencing [9] ADAM17 in human endothelial cells inhibited cell proliferation and prevented the formation of capillary networks in three-dimensional matrices [8]. Given the implication of ADAM17 activity in angiogenesis in vitro, we sought to address whether a catalytically inactive form of ADAM17 could impact vessel formation and function during embryonic development in vivo. We observed vascular malformations and hemorrhage during embryonic development of mice homozygous for a mutation inactivating ADAM17, thus demonstrating a role for this protease in modulating vascular development.

## Results

### ADAM17 Inactivation Causes Hemorrhaging in Developing Embryos

Intercrosses among heterozygous *Adam17* ^ΔZn/+^ mice were conducted to generate homozygous embryos deficient in ADAM17 activity. As previously reported [5], we observed an increased mortality of the *Adam17* ^ΔZn/ΔZn^ embryos at E17.5 (50% of embryos died, Supplementary Figure S1A) but not at earlier developmental stages (E11.5 and E14.5). Macroscopic observation at E14.5 and E17.5 revealed that *Adam17* ^ΔZn/ΔZn^ embryos showed a tendency to be underweight (Supplementary Figure S1B) and shorter in length (data not shown) as compared with the *Adam17* ^+/+^ and *Adam17* ^ΔZn//+^ fetuses although the differences were not statistically significant.

**Figure 1:**
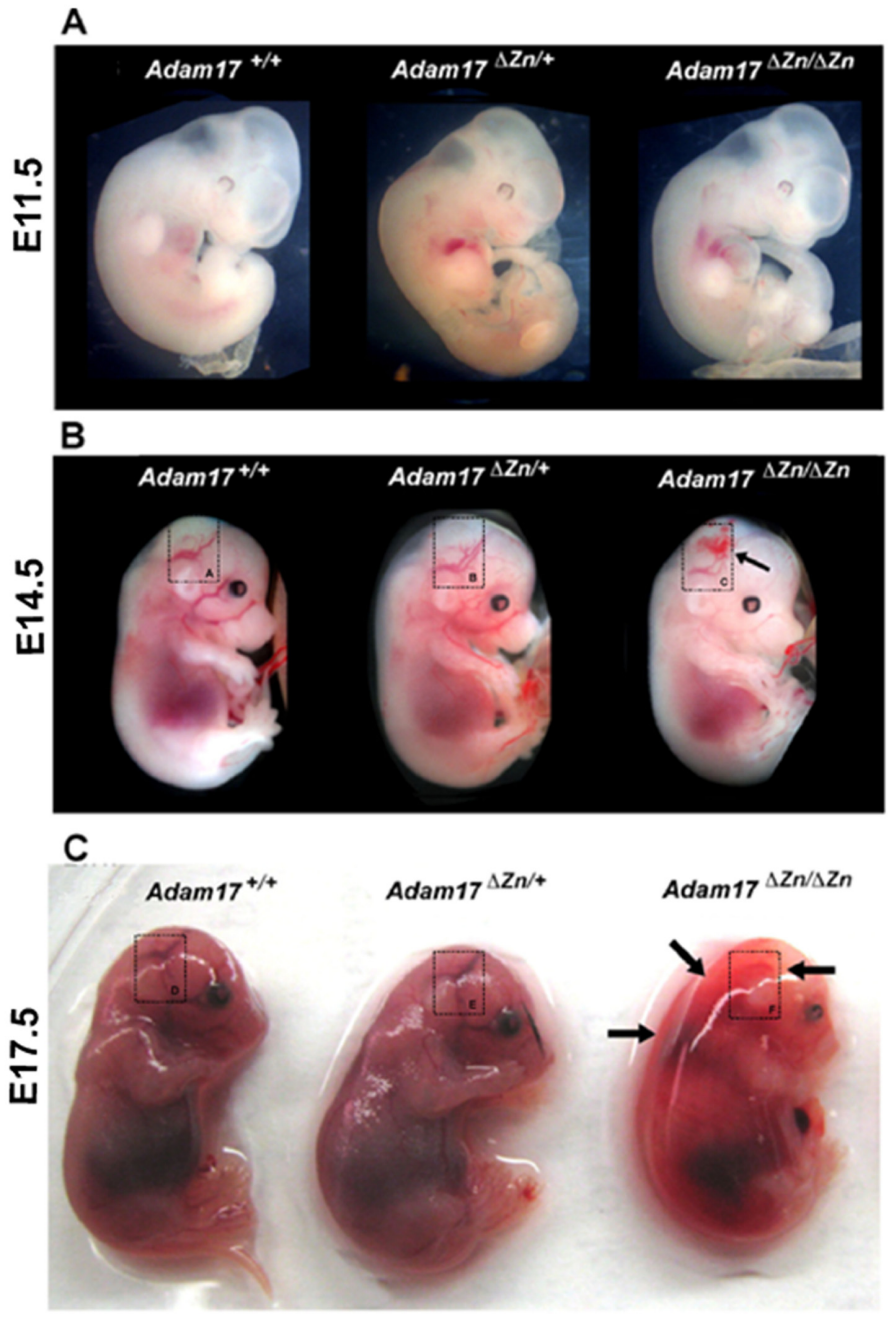
*Adam17* ^ΔZn/ΔZn^ embryos develop internal hemorrhage. Whole-mount views of *Adam17* ^+/+^, *Adam17* ^ΔZn/+^ and *Adam17* ^ΔZn/ΔZn^ embryos at E11.5 (A), E14.5 (B) and E17.5 (C); embryos were unfixed, unstained. *Adam17* ^ΔZn/ΔZn^ embryos exhibited hemorrhagic lesions compared with their *Adam17* ^+/+^ and *Adam17* ^ΔZn/+^ littermates (see dotted boxes in B and C and higher magnification views in Figure 2). Hemorrhage was observed in E14.5 embryos and increased with gestational age. At later stage E17.5, the arrows denote edema.

**Figure 2:**
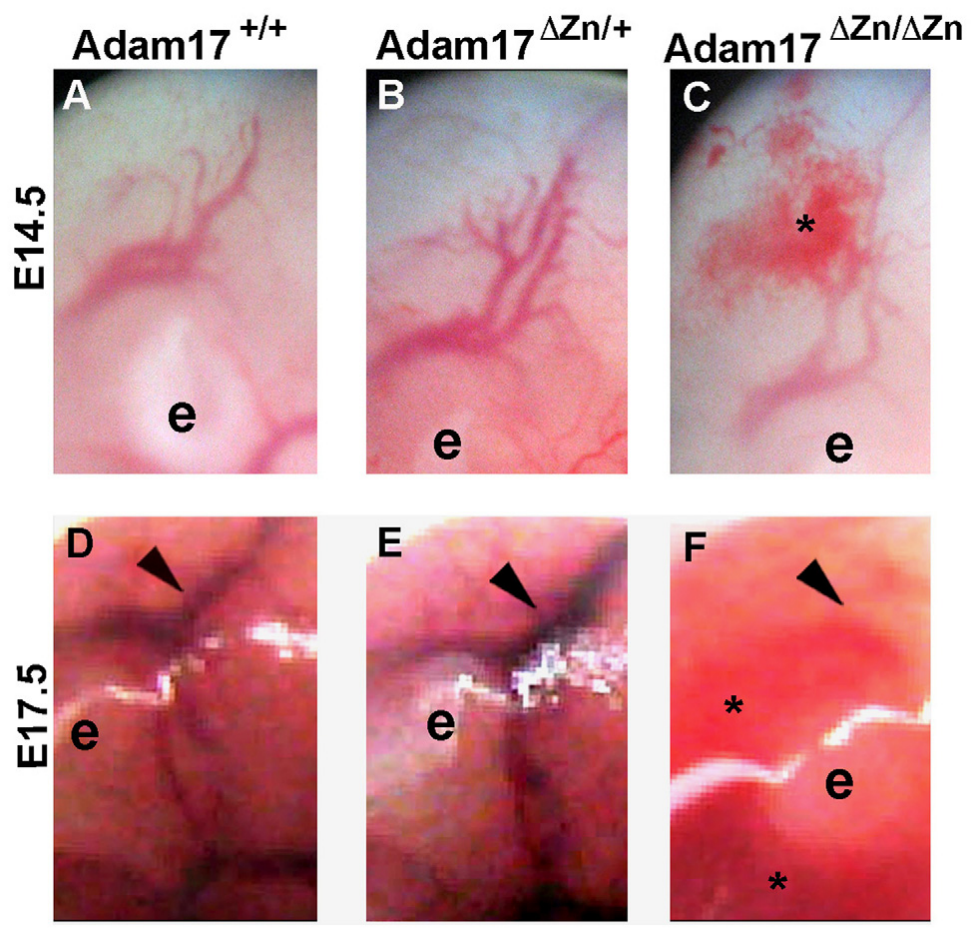
Vascular defects and hemorrhage in *Adam17* ^ΔZn/ΔZn^ embryos. Higher magnifications of the whole unfixed, unstained embryos at E14.5 (A, B, C) and E17.5 (D, E, F) as in Figure 1. *Adam17* ^ΔZn/ΔZn^ embryos (C, F) exhibited blood pooling (star) and loss of visible vascular structures (arrowheads); **e** = ear.

A detailed examination of the fetuses revealed that *Adam17* ^ΔZn/ΔZn^ embryos appeared to develop normally until E11.5 (Figure 1A). However at E14.5 and E17.5, 50% and 67% of the *Adam17* ^ΔZn/ΔZn^ embryos, respectively, exhibited internal hemorrhaging (Table 1). The *Adam17* ^+/+^ and *Adam17* ^ΔZn/+^ littermates did not show signs of bleeding at any developmental stage we studied (Table 1 and Figure 1). In the *Adam17* ^ΔZn/ΔZn^ embryos bleeding appeared between E11.5 and E14.5 and localized primarily on the side of the head of the embryos and the extent and localization of bleeding progressed with embryonic age (Figure 1B and 1C). Furthermore, at E17.5 the mutant embryos showed signs of edema on the neck and the upper back area. We found no hemorrhagic lesions on the yolk sac vasculature of the *Adam17* ^ΔZn/ΔZn^ embryos (data not shown). Upon closer examination we observed that at the late developmental stage (E17.5) blood pooling was accompanied by a loss of the vascular organization in mutant embryos compared with the heterozygous and wild-type littermates (Figure 2, compare arrows in D and E with F). These results suggest a defect in vessel integrity in the *Adam17* ^ΔZn/ΔZn^ embryos.

**Table 1:** Hemorrhagic Lesions in Littermates Derived from *Adam17* ^ΔZn/+^ Intercrosses.

	*Adam17* ^+/+^			*Adam17* ^ΔZn/+^			*Adam17* ^ΔZn/ΔZn^		
Developmental stage	E11.5	E14.5	E17.5	E11.5	E14.5	E17.5	E11.5	E14.5	E17.5
Embryos with hemorrhagic lesions	0	0	0	0	0	0	0	3	4
(% of n)	0%	0%	0%	0%	0%	0%	0%	50%	67%
n	7	10	5	14	15	12	5	6	6

### Impaired Vascular Patterning in Mutant Embryos

**Figure 3:**
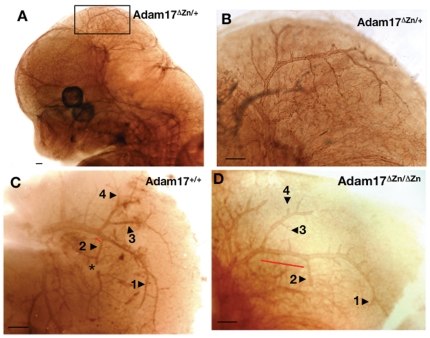
*Adam17* ^ΔZn/ΔZn^ vascular phenotype. Whole-mount E14.5 embryos were stained with CD31 (PECAM) antibodies to characterize their vascular morphology. Panel A shows the vasculature of an intact heterozygous embryo. Boxed area indicates the location of the region shown at higher magnification in panel B after dissection to allow flat mounting to view the cranial vessels. Similar views from heads of wild-type (C) and mutant (D) embryos. Wild-type patterning of the branches is severely disrupted in *Adam17* ^ΔZn/ΔZn^ embryonic heads. Vessels numbered 1–4 are stunted and less branched in the mutant embryos. Although the distance between vessel 2 and the branch leading up to vessels 3 and 4 seems increased in the mutant head shown (red bar), this distance was variable in wild-type heads. The asterisk marks a vessel damaged during dissection. Scale bars are 200 µm.

**Figure 4:**
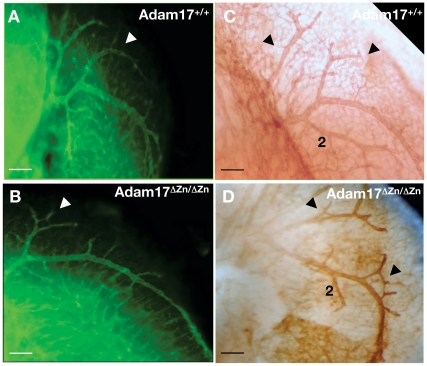
Abnormal fusion of branches in *Adam17* ^ΔZn/ΔZn^ cranial vessels. In addition to the stunted morphology, CD31 staining of E14.5 heads revealed abnormal joining of vessels at various branch points in the mutant embryos (3/4 embryos). Arrows indicate these fused branches in *Adam17* ^ΔZn/ΔZn^ embryos (B, D) and their absence in the wild-type counterparts (A, C). Scale bars are 200 µm.

To investigate the potential causes of bleeding in *Adam17* ^ΔZn/ΔZn^ embryos, we compared the vasculature of wild-type and mutant embryos as revealed by CD31 (PECAM) immunohistochemistry. Since the first evidence of hemorrhage was detected at around E14.5, we compared the whole-mount PECAM-1 (CD31) staining patterns of wild-type, *Adam17* ^+/ΔZn^ and *Adam17* ^ΔZn/ΔZn^ embryos at this stage (Figure 3 and 4). At this developmental period, there were no obvious defects in the general morphology of the mutant embryonic body vasculature compared with that of wild-type embryos. However, closer examination of the cranial vascular patterning revealed striking differences between the mutant embryos and their wild-type or heterozygous counterparts. At this stage, the large cranial vessels projecting posteriorly over the midbrain region showed an elaborate but reproducible branching pattern in heterozygous (Figure 3A, B) or wild-type embryonic heads (Figure 3C). In contrast, the development of this branched morphology was markedly disrupted in the *Adam17* ^ΔZn/ΔZn^ embryos (Figure 3D). For example, vessels numbered 1–4 in Figure 3 were longer and often further elaborated with additional branching in the wild-type embryos in contrast with the corresponding vessels in *Adam17* ^ΔZn/ΔZn^ embryos. In addition to this stunted vessel morphology, analysis of the *Adam17* ^ΔZn/ΔZn^ mutant phenotype revealed abnormal fusion of vessels in 3 out of 4 embryos (Table 2). This type of inappropriate joining of vessels was not restricted to a particular branch (Figure 4B and 4D) and was sometimes seen in multiple branches in the same embryo (Figure 4D). This defect was not observed in four wild-type embryos examined (Figure 4A, C). These vascular defects at E14.5 appeared to precede the hemorrhage and could well be contributory to later defects. The defects in vascular modeling implicate ADAM17 in this process, raising the question of potential targets for this cell surface protease.

**Table 2:** Descriptive Summary of the *Adam17* ^ΔZn/ΔZn^ Cranial Vasculature Phenotypes.

Vessel	*Adam17* ^+/+^	*Adam17* ^ΔZn/ΔZn^
1	One main branch point, with each vessel containing a single minor branch (4/4 embryos)	One main branch point, stunted vessel (4/4 embryos), minor branches absent (2/4 embryos)
2	Well-developed vessel with at least 1 branch point (4/4 embryos)	Stunted, non-branched (3/4 embryos), absent (1/4 embryos)
3	Well-developed (4/4 embryos) and branched (3/4 embryos)	Severely stunted (1/4 embryos) or absent (3/4 embryos)
4	Present (4/4 embryos), well-developed and branched (1/4 embryo)	Stunted (2/4 embryos) or absent (2/4 embryos)

## Discussion

Studies on the embryogenesis of mice deficient in ADAM17 activity reveal that these animals suffer from severe defects in the maturation and the differentiation of epithelial cells resulting in an impaired development of intestine, parathyroid, salivary glands lung and heart [5]–[7]. We now report that *Adam17* ^ΔZn/ΔZn^ embryos also present abnormal vascular beds that are probably responsible for internal hemorrhages appearing around E14.5. However, the vasculature of *Adam17* ^ΔZn/+^ heterozygous embryos develops normally, similarly to that of wild-type embryos, revealing that partial expression of ADAM17 is sufficient to obtain normal vascular development. We did not find major alterations in the initial steps of vascular development in *Adam17* ^ΔZn/ΔZn^ embryos but observed abnormal vessel branching patterns at the later stages of embryonic development compared with those seen in wild-type embryos. Associated with these vascular abnormalities, we also observed hemorrhage and edema. These observations are in accordance with a previous report where the authors detected a poorly developed capillary network in the lungs of these embryos [7]. Altogether these results suggest that ADAM17 activity seems not to be required for vasculogenesis or early angiogenesis but is necessary for proper remodeling of pre-existing vessels and the establishment of capillary networks. Reduction in the number of vessel branchpoints and in the capillary density is often linked with defects in the migratory properties of endothelial cells. Indeed, a role for ADAM17 in modulating endothelial cell sprouting and ability to invade was recently suggested in cultured human endothelial cells in which ADAM17 expression was silenced by delivery of either siRNA or dominant negative constructs [8], [9]. These in vitro data provide an interesting mechanistic hypothesis for a role of ADAM17 in endothelial sprouting capability that conforms with our in vivo observations.

The vascular defects we observed were likely intrinsic to the endothelium. We investigated pericyte coverage of the affected cranial vessels by staining for NG2 or α-smooth muscle actin. Neither stain detected any pericytes associated with the relevant vessels in either wild-type or mutant embryos although both stains readily detected pericytes around other vessels in the embryos at the same stage (data not shown). Therefore, the vascular defects and hemorrhage precede pericyte coverage of the affected vessels, suggesting that these vascular defects originate in the endothelium. Consistent with that conclusion, a recent paper, investigating an animal model of pathological angiogenesis [10] showed a role for ADAM17 in endothelial cells but not pericytes. Interestingly, in that paper no apparent defects in developmental angiogenesis were reported suggesting either that the developmental defects that we report can be overcome in subsequent development or that additional defects arise from absence of ADAM17 in other cell types. We did check for the possible involvement of cells of the monocyte/macrophage lineage by staining for F4/80 but no F4/80-positive cells were present in the region of the vessels in which we observed vascular defects (data not shown), arguing against a role for cells of this lineage.

Defects in angiogenesis are often accompanied by hemorrhage [11]. In normal angiogenesis, hemorrhage is prevented by platelets and their adhesive function through the von Willebrand factor receptor, GPIbα [12]. Platelets express ADAM17 that mediates shedding of GPIbα and several other platelet receptors after platelet activation. However, in chimeric mice expressing the inactive ADAM17 on blood cells only, produced by fetal liver transplant, the hemostatic function of platelets is unaffected or even improved as they cannot shed GPIbα [13]. Thus the observed hemorrhaging in the *Adam17* ^ΔZn/ΔZn^ embryos is not likely to be due to defective platelet function in these animals.α

Although the molecular mechanisms by which ADAM17 affects vessel development and branching still remain unknown, our data implicate ADAM17 enzymatic activity and not, as recently hypothesized, ADAM17 interaction with integrins or other proteins via its disintegrin domain [8]. In the *Adam17* ^ΔZn/ΔZn^ model, ADAM17 remains expressed in cells and is structurally unchanged except that its catalytic domain is genetically inactivated [5]. Despite the presence of the disintegrin domain, we detect vascular abnormalities in mutant embryos even though interactions with integrins could still occur. It is known that ADAM17 is responsible for the cleavage of the extracellular domains of numerous proteins that participate in the angiogenic process. Among these substrates is TNF-α (Tumor Necrosis Factor Alpha), a pleiotropic pro-inflammatory molecule, which also modulates angiogenesis. TNF-α was first described as an antiangiogenic compound causing tumor regression [14] but also exhibits proangiogenic properties in some in vivo conditions [15], [16]. The growth factor TGF-α (Transforming Growth Factor Alpha) is an ADAM17 substrate that was implicated in the epithelial abnormalities of *Adam17* ^ΔZn/ΔZn^ embryos and has also been shown to promote angiogenesis [17]. However, the vascular phenotype of *Adam17* ^ΔZn/ΔZn^ embryos' seems unlikely to be attributable to a loss of soluble TNF-α or TGF-α; to our knowledge, defects in embryonic vascularization or a bleeding phenotype have not been reported in mice expressing a non-cleavable form of TNF-α [18], [19] nor in animals deficient in TNF-α [20] or TGF-α [21]. However, there are other ADAM17 substrates that are clearly implicated in vascular development and represent likely candidates for an involvement in the phenotypes we report here. These include the receptors Notch [22], VEGFR-2 [23], and JAM-A [24] each of which can act at different levels of the angiogenic and remodeling processes [25]–[31].

In conclusion, our data indicate that ADAM17 enzymatic activity plays crucial roles in endothelial biology and in vascular development in vivo. Therefore, further investigations of potential ADAM17 substrates need to be undertaken to determine how ADAM17 shedding activity regulates blood vessel formation, remodeling and stability.

## Materials and Methods

### *Adam17* ^ΔZn/ΔZn^ Embryo Generation

*Adam17* ^ΔZn/+^ heterozygous mice (C57BL/6J/129Sv background) were kindly provided by Jacques Peschon, Amgen (Seattle, WA). The Adam17 gene was mutated by deletion of the zinc-binding site through homologous recombination [5]. Homozygous *Adam17* ^ΔZn/ΔZn^, *Adam17* ^ΔZn/+^ and *Adam17* ^+/+^ embryos were produced by intercrossing *Adam17* ^ΔZn/+^ heterozygous mice on a C57BL/6J/129Sv background and the resulting littermates were used for analysis. Day 0.5 of pregnancy (E0.5) was defined as the day when a vaginal plug was observed. Animal protocols were approved by the Animal Care and Use Committee of the Immune Disease Institute (IDI4M0109/HMS04564). The Immune Disease Institute's Animal Care Management Program is accredited by the American Association for the Accreditation of Laboratory Animal Care International and meets National Institutes of Health standards. The Institution also accepts as mandatory the PHS Policy on Humane Care and Use of Laboratory Animals by Awardee Institutions and NIH Principles for the Utilization and Care of Vertebrate Animals Used in Testing, Research, and Training. An approved Assurance of Compliance is on file with the Office of Laboratory Animal Welfare. The Immune Disease Institute's assurance number is A3251.

### *Adam17* ^ΔZn/ΔZn^ Embryo Genotyping

Genotyping was performed by PCR using genomic DNA extracted from paw tissue. Primers used were: *Adam17^+^* fwd 5′-CACGGGTAGCCAACGCTATGT-3′ and rev 5′-GCCCTGAATGAACTGCAGGACC-3′ ; *Adam17* ^ΔZn^ fwd 5′-CTTATTATTCTCGTGGTCACCGCT-3′ and rev 5′-GAAGCTGACCTGGTTACAACTCATG-3′.

### Body Weight Determination and Microscopy of Unfixed Embryos

Embryos were harvested from pregnant females at indicated time points and individually placed on a clean container for weight determination. The excess of fluid was removed by quick wicking on a gauze pad prior to weighing. Embryos were then observed and photographed under a Zeiss Stemi 2000 stereomicroscope.

### Immunohistochemistry

E14.5 embryos were fixed in methanol/DMSO (4∶1) at 4°C overnight. Following fixation, embryos were stored in 100% methanol at −35°C until further processing. The remaining staining procedure was done at room temperature unless indicated otherwise. For immunohistochemistry, endogenous peroxidase activity was quenched by incubating embryos with 2% hydrogen peroxide (H_2_O_2_) in methanol/DMSO (4∶1) for 4 hours. After the H_2_O_2_ treatment, embryos were washed in 75%, 50%, 25% methanol 15 minutes each. Washes with the methanol series were followed by three 15-minute rinses and an overnight incubation in PBS (without Ca/Mg). Embryos were then blocked in 10% normal goat serum (NGS) in PBS containing 0.1% TritonX-100 (PBST) for 2 hours. Blocked embryos were incubated overnight with primary rat antibody against CD31 (PECAM clone MEC13.3, BD Biosciences, San Jose, CA) diluted 1∶100 in blocking solution. Primary antibody incubation was followed by 6 one hour and one overnight washes in PBST. The overnight wash was then replaced with horseradish peroxidase (HRP)-conjugated anti-rat secondary antibody (Jackson ImmunoResearch, West Grove, PA) diluted 1∶50 in blocking solution. Embryos were incubated with the secondary antibody for two days, washed 6 times one hour each and overnight with PBST. HRP signal was developed with a DAB substrate kit (Vector Labs, Burlingame, CA) according to the manufacturer's instructions, except that PBST was substituted for the buffer provided. Developed embryos were post-fixed in 4% paraformaldeyde in PBS overnight at 4°C, washed 3 times with PBS and cleared by incubating in 50%, followed by 80% glycerol in PBS overnight at 4°C. Heads of stained embryos were dissected with electrolytically sharpened tungsten needles and mounted in 80% glycerol. Digital images of mounted heads were captured either with Openlab software using a Zeiss Axiophot microscope fitted with a Retiga Exi camera or with Spot software using a Zeiss Stemi 2000-C microscope fitted with a SpotRT Slider camera. Fluorescent staining of embryos was carried out as described above with some modifications. The NG2 antibody (Abcam, Cambridge MA) and PECAM antibody (clone MEC13.3, BD Biosciences, San Jose, CA) were diluted 1∶100 for the whole mount stains. The H_2_O_2_ treatment step was omitted and a fluorescein-conjugated secondary antibody (Invitrogen-Molecular Probes, Carlsbad, CA) was substituted for the HRP-conjugated anti-rat secondary antibody. After the secondary antibody incubation, the fluorescently labeled embryos were washed and incubated in mounting medium (CFM-1 Plus mounting medium, Electron Microscopy Sciences, Hatfield, PA) overnight at 4°C without post-fixation for dissections and mounting.

### F4/80 Staining of Paraffin-Embedded Tissue Sections

E14.5 wild-type embryos were fixed with 4% paraformaldehyde overnight. Embryos were washed with PBS (without Ca/Mg) and stored in 70% ethanol until processed for sectioning. Paraffin-embedded sections were de-waxed into water and heat-mediated antigen retrieval was performed using Retrievagen A kit (BD Biosciences, Franklin Lakes, NJ). Sections were blocked with 20% normal goat serum, 1% fish oil in PBT and for 2 hours at room temperature. Primary rabbit anti-PECAM (Abcam, Cambridge, MA) and rat anti-F4/80 (AbD Serotec, Raleigh, NC) antibodies diluted in 4% normal goat serum/PBT were incubated with the sections overnight at room temperature. Following washes with PBS, sections were incubated with fluorescent conjugated secondary antibodies (Invitrogen-Molecular Probes, Carlsbad, CA). After 6 washes with PBS, sections were mounted in CFM-1 Plus mounting medium (Electron Microscopy Sciences, Hatfield, PA) and imaged with a Zeiss Axiophot microscope.

### Statistics

Values are expressed as mean ± SEM. The statistical significance was assayed using the one-way ANOVA analysis of variance followed by Boneferroni's multiple comparisons test. A p value<0.05 was considered statistically significant.

## Supporting Information

Figure S1 Genotyping and weight of progeny derived from Adam17^ΔZn/+^ intercrosses. A, The number of viable embryos of each genotype is listed and the number of additional dead fetuses is indicated in parentheses. B, The weights of the embryos of each genotype were measured at E14.5 and E17.5; no statistical differences were observed among the different genotypes. Values are represented as mean ± SEM.
(2.23 MB EPS)
